# Macrophage variants in laboratory research: most are well done, but some are RAW

**DOI:** 10.3389/fcimb.2024.1457323

**Published:** 2024-10-09

**Authors:** Marc Herb, Valentin Schatz, Karina Hadrian, Deniz Hos, Bohdan Holoborodko, Jonathan Jantsch, Natascha Brigo

**Affiliations:** ^1^ Institute for Medical Microbiology, Immunology and Hygiene, Faculty of Medicine and University Hospital Cologne, University of Cologne, Cologne, Germany; ^2^ Department of Ophthalmology, Faculty of Medicine and University Hospital Cologne, University of Cologne, Cologne, Germany; ^3^ Institute of Clinical Microbiology and Hygiene, University Hospital Regensburg and University of Regensburg, Regensburg, Germany

**Keywords:** macrophage types, primary macrophages, macrophage-like cell lines, immunity, macrophage polarization (MP)

## Abstract

Macrophages play a pivotal role in the innate immune response. While their most characteristic function is phagocytosis, it is important not to solely characterize macrophages by this activity. Their crucial roles in body development, homeostasis, repair, and immune responses against pathogens necessitate a broader understanding. Macrophages exhibit remarkable plasticity, allowing them to modify their functional characteristics in response to the tissue microenvironment (tissue type, presence of pathogens or inflammation, and specific signals from neighboring cells) swiftly. While there is no single defined “macrophage” entity, there is a diverse array of macrophage types because macrophage ontogeny involves the differentiation of progenitor cells into tissue-resident macrophages, as well as the recruitment and differentiation of circulating monocytes in response to tissue-specific cues. In addition, macrophages continuously sense and respond to environmental cues and tissue conditions, adjusting their functional and metabolic states accordingly. Consequently, it is of paramount importance to comprehend the heterogeneous origins and functions of macrophages employed in *in vitro* studies, as each available *in vitro* macrophage model is associated with specific sets of strengths and limitations. This review centers its attention on a comprehensive comparison between immortalized mouse macrophage cell lines and primary mouse macrophages. It provides a detailed analysis of the strengths and weaknesses inherent in these *in vitro* models. Finally, it explores the subtle distinctions between diverse macrophage cell lines, offering insights into numerous factors beyond the model type that can profoundly influence macrophage function.

## 1 Macrophages

Macrophages represent an ancient cell type in the phylogeny of metazoans (Wittamer et al., 2011; Buchmann, 2014). They are capable of engulfing foreign or endogenous materials and are found in many organisms with multicellular organization, where they have versatile roles in maintaining homeostasis and immunological defense (Mosser and Edwards, 2008; Wynn et al., 2013; Barreda et al., 2016; Mosser et al., 2021). They were named (“makros” = big, “phagein” = to eat, “big eaters”) after their most characteristic ability and main function, the active uptake of particles bigger than 0.5 µm, by a process called phagocytosis, firstly discovered by Metchnikoff in the beginning of 1880 (Underhill and Goodridge, 2012; Tauber et al., 2017).

### 1.1 Phagocytosis: a crucial cellular mechanism in macrophages for immune defense and tissue homeostasis

Phagocytosis is an important cellular mechanism conserved in all multicellular organisms from protozoans to mammals, including humans (Boulais et al., 2010; Gordon, 2016). Macrophages phagocytose endogenous material, like apoptotic cells (Fadok et al., 1998; Liu et al., 2006; Erwig and Henson, 2008; Kono and Rock, 2008; Suzanne and Steller, 2013; Kourtzelis et al., 2020) and cell debris, or foreign objects, such as pathogens (Chen et al., 2007; Gluschko et al., 2018) and toxic substances, like asbestos or silica particles (Murray and Wynn, 2011b; Nakayama, 2018).

Macrophages play a vital role in engulfing and digesting particles through phagocytosis, a function that aids in identifying different subtypes within the macrophage classification based on how efficiently and extensively they perform this process (Ku et al., 1992; Kirkpatrick et al., 1997; Neve et al., 2014; Kresinsky et al., 2016).

For scanning their extracellular surroundings, macrophages express numerous surface and cytoplasmic receptors that detect irregular signals not typically found in the organism’s physiological milieu (Uribe-Querol and Rosales, 2020). These include scavenger receptors, which bind apoptotic and necrotic cells, opsonized pathogens, and cell debris; and pattern recognition receptors (PRRs) that detect ‘non-self’ or ‘damaged’ signals (Kawai and Akira, 2011), such as Toll-like receptors (TLRs), C-type lectin receptors (CLRs), retinoic acid-inducible gene 1 (RIG1)-like helicase receptors (RLRs), Fc‐receptors, and NOD-like receptors (NLRs) (Elinav et al., 2011; Osorio and Reis e Sousa, 2011; Freeman and Grinstein, 2014; Uribe-Querol and Rosales, 2020).

The interaction of these receptors with ligands recruits actin filaments to internalize the particle, which is then enclosed within a phagosome (Sarantis and Grinstein, 2012; Weiss and Schaible, 2015). Phagosomes fuse with lysosomes to form phago-lysosomes, leading to the degradation of the cargo (Jutras and Desjardins, 2005; Haas, 2007; Levin et al., 2016).

### 1.2 Efferocytosis: macrophages’ strategy for infection control and tissue maintenance

Macrophages can contain the spread of infection through a clearance of infected and dead host cells (Martin et al., 2012). This behavior is called efferocytosis and serves as a protective mechanism to clear dying or dead cells from tissues during growth and remodeling (Boada-Romero et al., 2020). This process is primarily carried out by tissue macrophages and, during the onset of inflammation, by monocyte-derived macrophages (Watanabe et al., 2019).

In instances of infection, a significant number of cells engaged in host defense undergo cell death. It becomes imperative to remove these cells to minimize tissue damage and inflammation (Labbe and Saleh, 2008). Additionally, since these cells are often harboring intracellular pathogens, which lose their habitats due to cell death, containing the infection becomes critical, rendering efferocytosis an indispensable process during the host’s response to intracellular bacteria (Weiss and Schaible, 2015; Karaji and Sattentau, 2017). In addition, efferocytosis promotes the transition of macrophages to the anti-inflammatory phenotype, leading to a reduction in pro-inflammatory cytokines and an increase in the release of anti-inflammatory mediators, such as interleukin (IL)-10 and transforming growth factor-beta (TGF-β) and pro-resolving molecules (resolvins and protectins) (Dalli and Serhan, 2012; Angsana et al., 2016; Ge et al., 2022), which aid in the reduction of inflammation during infection (Elliott et al., 2017; Chazaud, 2020).

Moreover, efferocytosis plays a crucial role in tissue restructuring during growth and development, as well as in the process of wound healing (Vaught et al., 2015; Mehrotra and Ravichandran, 2022). Throughout the development of complex organisms, cell death is a natural occurrence that aids in growth and tissue restructuring. Thus, eliminating senescent and deceased cells becomes vital for preserving tissue balance and structure, while also facilitating the healing process (Morioka et al., 2019; Gerlach et al., 2021).

### 1.3 Optimizing macrophage function: the crucial role of the extracellular matrix and fibroblast interactions

Ensuring a favorable environment for macrophages is important for their optimal function as phagocytes (Chen et al., 2023). The extracellular matrix (ECM) serves as a sophisticated system that offers a structural scaffold supporting immune cells. Particularly, collagen, predominantly synthesized by fibroblasts, plays a key role in facilitating various cellular functions, including the differentiation and adhesion of myeloid cells (Khalaji et al., 2017; Makdissi and Mass, 2021). In addition, the interplay of macrophages and fibroblasts is important for growth factor exchange and reducing abnormal proliferation (Zhou et al., 2018). Finally, modification of ECM can trigger a mechanosensitive response in macrophages which is critical for tissue regeneration and fibrosis (Meizlish et al., 2024).

As the origin of macrophages, whether isolated from animal or human samples, differentiated with growth factors from precursor cells, isolated as cancer cell lines exhibiting macrophage-like behavior, or generated as *in vitro* cell lines, understanding alterations in macrophage behavior solely based on its origin is futile. Moreover, characterizing macrophages by their phagocytic activity needs to be expanded, as macrophage function during processes such as body development, homeostasis, repair, and immune responses against invading pathogens is of paramount importance.

## 2 Macrophage development: diverse origins and functional adaptations

As macrophages are pivotal components of the innate immune system, their development is tightly regulated in a series of stages within the hematopoietic system (Wynn et al., 2013). For many years, there was a prevailing belief that macrophages constituted a largely uniform group originating from hematopoietic stem cells (HSCs) in the bone marrow and underwent a defined developmental process known as monopoiesis (Mosser and Edwards, 2008). However, recent findings show that numerous macrophages residing in tissues of adult mice do not depend on HSCs, instead, they originate from yolk-sac progenitors in early embryonic stages (Gomez Perdiguero et al., 2015).

These precursor cells, termed pre-macrophages (pMacs), travel through the bloodstream and settle in various organs. Upon exiting the bloodstream and undergoing differentiation, they develop a unique genetic profile specific to the tissue they inhabit (Mass, 2023; Wang et al., 2023). This maturation process equips macrophages with the ability to adapt to particular tissue contexts (Gordon and Pluddemann, 2017). The resulting macrophages can assume diverse roles, ranging from resident tissue macrophages contributing to homeostasis or to activated macrophages responding dynamically to infections or inflammatory stimuli (Gomez Perdiguero et al., 2015). Tissue-resident macrophages possess the ability to renew themselves and typically do not rely on input from HSCs (Mass et al., 2023).

Furthermore, each organ contains monocyte-derived macrophages (MDMs) with varying lifespans, some persisting long-term while others have shorter lifespans and are continuously replaced by HSCs from the bone marrow. This leads to a complex situation where macrophages originating from fetal and HSC sources coexist in certain tissues (Jenkins and Allen, 2021; Dick et al., 2022).

At the site of inflammation, monocytes are attracted to the tissue and differentiate into macrophages, which cooperate with or replace resident cells for sustaining immunity or promoting resolution of inflammation and tissue regeneration (Italiani and Boraschi, 2015; Muller et al., 2020; Mass et al., 2023).

### 2.1 Tissue macrophages

Tissue macrophages show remarkable functional diversity and plasticity, which are influenced by the surrounding tissue (Sreejit et al., 2020; Mass et al., 2023). Studies employing fate-mapping techniques involving conditional reporter genes have indicated that most resident tissue macrophage populations are established during embryonic development and sustain themselves in adulthood through self-renewal, with limited contribution from bone marrow progenitors or blood monocytes (van Furth and Cohn, 1968; Hume, 2023). Tissue macrophage progenitors (for skin, spleen, pancreas, liver, brain) are derived from yolk sac and fetal liver (Bertrand et al., 2005; Gomez Perdiguero et al., 2015) or have mixed origins from yolk sac and bone marrow (for lung, kidney) (Hoeffel et al., 2012; Epelman et al., 2014).

Some macrophage populations, that are characterized by low F4/80 receptor expression, can be replenished from bone marrow-derived progenitor cells under certain circumstances, such as tissue infection (Ginhoux et al., 2010; Hoeffel et al., 2012; Schulz et al., 2012). In their basal state, resident tissue macrophages exhibit considerable variation in function, requiring different morphologies, transcriptional responses, and locations (Davies and Taylor, 2015). This functional heterogeneity probably results from the dynamic crosstalk between resident tissue macrophages and client cells that they support (de Sousa et al., 2019).

**Figure 1:**
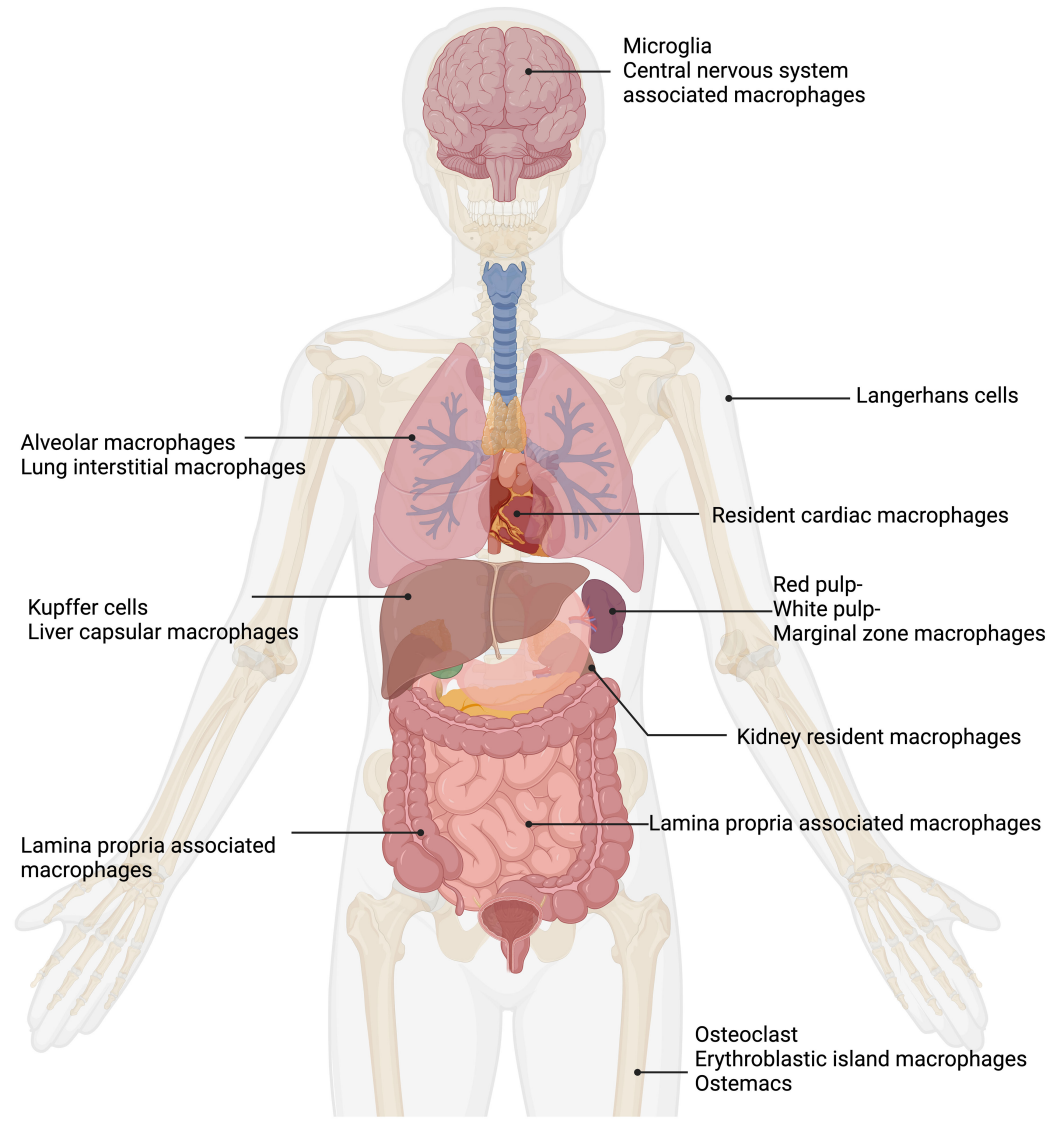
Tissue macrophages exhibit remarkable functional diversity depending on their tissue of residence. In the central nervous system (CNS), microglia (Pallares-Moratalla and Bergers, 2024) and CNS-associated macrophages (Prinz et al., 2021) reside. In the skin, tissue-resident macrophages are Langerhans cells (Merad et al., 2002). The lung contains two types of tissue-resident macrophages: alveolar macrophages (Joshi et al., 2018) and lung interstitial macrophages (Schyns et al., 2018). Tissue-resident macrophages of the spleen are divided into red pulp, white pulp, and marginal zone macrophages (Fujiyama et al., 2019). Resident cardiac macrophages critically contribute to heart tissue remodeling (Wong et al., 2021; Suku et al., 2022). Kidney resident macrophages support organ healing (Cheung et al., 2022). The liver comprises two main types of tissue-resident macrophages: Kupffer cells (Dixon et al., 2013) and liver capsular macrophages (Bleriot and Ginhoux, 2019). The gut contains lamina propria-associated macrophages (Nagashima et al., 1996). In the bone, three types of tissue-resident macrophages are present: osteoclasts (Drissi and Sanjay, 2016), erythroblastic island macrophages (Seu et al., 2017), and osteomacs (Batoon et al., 2017). Figure was adapted from the template “Human Internal Organs” by Eunice Huang. Retrieved from https://app.biorender.com/biorender-templates; Biorender.com.

The roles of tissue resident macrophages have been reviewed (Davies et al., 2013a; Epelman et al., 2014; Davies and Taylor, 2015; Varol et al., 2015; Lazarov et al., 2023). Macrophages are divided into subpopulations based on their anatomical location and functional phenotype (**Figure 1**). A remarkably large reservoir of tissue resident macrophages lies in the intestine (Gordon et al., 2014).

Interestingly, it is believed that murine intestinal tissue-resident macrophages are regularly replenished by circulating monocytes via a phenomenon referred to as the “monocyte waterfall” (Bsat et al., 2020; Muller et al., 2020). Embryonic precursors colonize the intestinal mucosa and undergo significant proliferation *in situ* during the neonatal phase. However, they are replaced around weaning by Ly6C^high^ monocytes. These monocytes then mature into anti-inflammatory macrophages locally. This process, primarily influenced by the microbiota, needed to persist throughout adulthood to uphold a healthy population of intestinal macrophages (Bain et al., 2014; Desalegn and Pabst, 2019).

Tissue macrophages contribute to the maintenance of healthy tissues by removing dead and dying cells, as well as toxic materials (Murray and Wynn, 2011b). Tissue macrophages also suppress inflammation mediated by inflammatory monocytes, thereby ensuring that tissue homeostasis is restored after infection or injury. Indeed, important homeostatic functions have been assigned to the mononuclear phagocytes in almost every tissue of the body (Murray and Wynn, 2011b; Wynn et al., 2013; Chovatiya and Medzhitov, 2014; Davies and Taylor, 2015; Mosser et al., 2021).

## 3 Macrophage plasticity and polarization: beyond the M1/M2 paradigm

Macrophages display an incredible plasticity, where their functions can be significantly and specifically altered by cytokine signals (Murray and Wynn, 2011b; Wynn et al., 2013). Due to their ability to change and adapt to various exogenous and endogenous factors, macrophages can rapidly alter their functional profile through a process known as polarization (Mosser et al., 2021). Macrophage polarization is the process by which macrophages respond to incoming stimuli from the local microenvironment (Murray, 2017; Arora et al., 2018). After recognition of the phagocytosed material and/or additional factors, such as secreted pro- or anti-inflammatory cytokines from other cells, several signaling pathways are activated. These pathways ultimately lead to the development of a specific macrophage phenotype tailored to meet the functional requirements (Stout et al., 2005).

Currently, there is a partial consensus among researcher in the macrophage field concerning the classification of various macrophage activation phenotypes (Martinez and Gordon, 2014; Ginhoux et al., 2016; Murray, 2017). Initially, macrophage activation relied on a simplistic binary model, such as classic and alternative activation concepts, also known as M1 and M2. It is now widely acknowledged that this model is overly broad and misinterpreted, hindering the understanding of pathogenesis (Martinez and Gordon, 2014; Muraille et al., 2014).

Classically activated macrophages (or M1-like macrophages) release a plethora of pro-inflammatory cytokines, and produce antimicrobial factors, like reactive nitrogen species (RNS) or reactive oxygen species (ROS) (Nathan et al., 1983; Filipe-Santos et al., 2006; Mosser and Edwards, 2008; O’Shea and Murray, 2008; Orecchioni et al., 2019; Dalby et al., 2020; Herb and Schramm, 2021). Alternatively activated macrophages (or M2-like macrophages) have anti-inflammatory functions and regulate wound healing and tissue repair (Stein et al., 1992; Doyle et al., 1994; Loke et al., 2002; Kreider et al., 2007; Wynn et al., 2011; Sica and Mantovani, 2012; Corliss et al., 2016; Wynn and Vannella, 2016; de Groot and Pienta, 2018). There are in addition many subtypes of M2-like macrophages depending on the anti-inflammatory stimuli: M2a, M2b, M2c, M2d (Mantovani et al., 2004).

The currently prevailing perspective on macrophage activation involves adopting a multidimensional model that incorporates the diverse signals encountered by macrophages within their unique microenvironments i.e.: M(IL-4), M(IL-13), M(IFN-γ), M(LPS). This system sidesteps the intricacies of classifications, where different laboratories might define activation differently, enabling comparisons and contrasts of new activation conditions with these fundamental examples (Murray et al., 2014; Ginhoux et al., 2016).

Macrophages demonstrate remarkable phenotypic plasticity by rapidly modulating their functional capability in response to exogenous factors, such as infection or injury (Mosser and Edwards, 2008). These immune effectors are extremely dynamic, initially engaging in pro-inflammatory activities and subsequently transitioning to facilitate the resolution phase. This flexibility allows macrophages to regulate immune responses, from inflammation to restoring tissue balance (Mills, 2012; Gordon and Martinez-Pomares, 2017).

Taken together, there is not “the macrophage” per se, but a broad set of different macrophage types that constantly detect and respond to environmental stimuli as well as tissue physiology and change their functional and metabolic state accordingly (Nathan et al., 1983; Pace et al., 1983; Stein et al., 1992; Doyle et al., 1994; Mills and O’Neill, 2016; O’Neill and Pearce, 2016; Formentini et al., 2017). Having said this, it comes with no surprise that there cannot be a one fit all model for studying macrophage biology but that careful consideration is required upfront.

## 4 Macrophage colony-stimulating factor and its role in macrophage differentiation and cultivation

Macrophage colony-stimulating factor (M-CSF) is a growth factor responsible for the proliferation and differentiation of myeloid progenitors into macrophages in the bodies of humans and mice (Lin et al., 2008; Hamilton and Achuthan, 2013; Roszer, 2018; Hamidzadeh et al., 2020).

In the plasma of mice, approximately 10 ng/mL M-CSF is constantly present, maintained by secretion from various cells in the body, thereby ensuring the recruitment and differentiation of circulating blood monocytes (Hume and MacDonald, 2012; Hamilton and Achuthan, 2013). Under physiological conditions, M-CSF levels are regulated by colony stimulating factor 1 receptor (CSF-1R)-mediated endocytosis, providing feedback control to regulate macrophage production based on the number of mature macrophages (Bartocci et al., 1987). A key characteristic of *in vivo* macrophages is their dependence on M-CSF receptor signaling for differentiation and survival (Ushach and Zlotnik, 2016).

M-CSF is frequently utilized *in vitro* to generate bone marrow-derived macrophages (BMDM) from myeloid progenitor cells in the bone marrow of mice and to differentiate macrophages from peripheral blood monocytes (PBMC) for human-related research (Ushach and Zlotnik, 2016). These cells need M-CSF for both, differentiation and survival *in vitro* (Erbel et al., 2013; Chen et al., 2021b). In contrast, *ex vivo* macrophages, such as peritoneal macrophages, are isolated as mature macrophages and therefore do not need M-CSF for further differentiation. However, they only survive 2-3 days during *in vitro* cultivation without M-CSF supplementation (Wang et al., 2013).

In sharp contrast to *in vitro*-differentiated or *ex vivo*-cultivated macrophages, the macrophage-like cell lines RAW264.7 and J774 do not need M-CSF or any other constantly applied growth factor for cultivation or survival *in vitro* (Ralph and Nakoinz, 1975; Raschke et al., 1978).

Islam et al. reported minimal production of M-CSF in RAW264.7 cells, which was significantly increased due to receptor activator of nuclear factor kappa B (NF-κB) ligand (RANKL) treatment (Islam et al., 2008). Another study on osteoclast formation from the macrophage-like cell line RAW264.7 stated that the immortalized cell line produces M-CSF (Collin-Osdoby and Osdoby, 2012). Moreover, M-CSF levels in the supernatant of RAW264.7 cells were detected via ELISA in another study (Yang et al., 2014).

Checking available high-throughput RNA-sequencing data, two publications showed the expression of M-CSF in RAW264.7 cells (Chen et al., 2021a; Liu et al., 2023a). The levels of M-CSF significantly increased due to stimulation with lipopolysaccharide (LPS) (Liu et al., 2023a) and decreased due to the infection with *Mycobacterium tuberculosis* (*Mtb*) (Chen et al., 2021a). One publication by Andreu et al. showed that J774 cells express higher levels of the gene for M-CSF compared to BMDM. The level of M-CSF increased over time and in response to infection with *Mtb* in J774 cells (Andreu et al., 2017). The published results of the RNA-sequencing data are summarized in **Table 1**.

**Table 1:** M-CSF expression in macrophage-like cell lines and bone marrow-derived macrophages.

Cell Type	Treatment	Avg. counts	Counts relative to LPS	Reference
RAW264.7	untreated (6 h)	103.3	49.3	Liu et al., 2023a
RAW264.7	500 ng/mL LPS (6 h)	5097.3		
			Counts relative to *Mtb* infection	
RAW264.7	untreated (3 h)	142.0	0.4	Chen et al., 2021a
RAW264.7	*Mtb* infection (3 h)	57.0		
			Counts relative to BMDM	
BMDM	untreated (4 h)	14.7	3.4	Andreu et al., 2017
J774	untreated (4 h)	49.3		
BMDM	*Mtb* infection (4 h)	543.7	0.6	
J774	*Mtb* infection (4 h)	309.0		
BMDM	untreated (24 h)	29.3	2.6	
J774	untreated (24 h)	76.3		
BMDM	*Mtb* infection (24 h)	123.3	6.7	
J774	*Mtb* infection (24 h)	827.7		

**Table 1** shows the total number of aligned reads for the M-CSF gene (raw counts; not shown) for each biological replicate was taken from the published data and used to estimate M-CSF expression by averaging (Avg. counts). The relative M-CSF expression was determined by forming a ratio between J774 and BMDM, RAW264.7 untreated and LPS stimulated or with *Mtb* infected samples. Independent filtering of the DESeq2 R package confirmed that all values have passed the filter threshold for low expressed genes (not shown).

However, only RNA data has been published and no study has reported M-CSF secretion in the macrophage cell line J774. Therefore, it remains unclear whether J774 cells produce their own M-CSF and thus do not require supplementation, or if this cell line simply does not need M-CSF due to their inherent proliferative capabilities as tumor-derived cells.

## 5 Immortalized and primary mouse macrophage models for *in vitro* studies

In general, immortalized macrophages derived from tumor cells, which exhibit continuous division or cells that have been deliberately modified to proliferate indefinitely, enable their cultivation over numerous generations (Taciak et al., 2018; Lucy et al., 2022; Chalak et al., 2024; Xie et al., 2024). Nonetheless, primary macrophages are the mainstay of study of macrophage function *in vitro* (Murray and Wynn, 2011a).

Each *in vitro* macrophage model has both positive and negative aspects that need to be carefully considered before choosing a model.

### 5.1 Immortalized mouse macrophage cell lines

The development of tissue culture techniques and establishment of macrophage cell lines has been indispensable for biological research for several decades (Freshney; Ralph et al., 1980; Sumiya et al., 2015; Taciak et al., 2018). Macrophage-like cell lines are important tools to unravel macrophage function, with RAW264.7 and J774 cells as the two most commonly used immortalized macrophage cell lines available from cell banks (Lam et al., 2009; Taciak et al., 2018). Immortalized macrophage cell lines have several advantages: (I) they are easy to handle and self-replicate, thereby representing an unlimited source of cells with various genetic conditions. (II) The cells can be cultivated in almost limitless quantities, (III) they can be stored frozen for a long time, and (IV) they are easily replaced if lost due to e. g. contamination (Kaur and Dufour, 2012; Voloshin et al., 2023; Weiskirchen et al., 2023).

However, there are a couple of limitations if immortalized macrophage cell lines are used. They are derived either from cancerous single cells/tumors or were generated by viral infection. That is why they are susceptible to genotypic and phenotypic drift during culture and passaging. Consequently, macrophage cell lines can lose macrophage-specific functions and acquire a molecular phenotype that is quite different from that of cells *in vivo* or primary isolated cells (Burdall et al., 2003; Pan et al., 2009; Frattini et al., 2015; Bosshart and Heinzelmann, 2016).

For example, RAW264.7 macrophages are lacking apoptosis-associated speck-like protein containing a caspase activation and recruitment domain (ASC) which serves as adaptor molecule for various inflammasome receptors (Pelegrin et al., 2008). This hinders RAW264.7 cells to produce and secrete mature IL-1β (while the production of pro-IL-1β is unaffected) after stimulation with nigericin for NLR family pyrin domain containing 3 (NLRP3), double-stranded DNA for absent in myeloma 2 (AIM), and *Clostridioides difficile* toxin b (TcdB) for pyrin inflammasome activation (Zheng et al., 2020). Transfection of RAW264.7 macrophages with ASC subunit restores the ability of pro-caspase 1 cleavage with subsequent production and secretion of mature IL-β (Bryan et al., 2010). In addition, RAW264.7 macrophages have fluctuating expression of several genes and proteins with increasing passage numbers. More than 50 passages increased the gene expression of hypoxia-inducible factor 1-alpha (*Hif1a)*, integrin subunit alpha L (*Itgal)*, cluster of differentiation 86 (*Cd86)*, and others in RAW264.7 macrophages while the expression of arginase 1 (*Arg1)*, transferrin receptor 2 (*Trf2)*, and interferon regulatory factor 8 (*Irf8)* changed already after 15 passages (Taciak et al., 2018).

Moreover, transfection with DNA, which is possible in RAW264.7 cells, but induces cell death in primary macrophages, suggests that this cell line is not comparable to primary macrophages (Hornung et al., 2009; Roberts et al., 2009; Liu and Guan, 2018; Paludan et al., 2019). Notably, J774 cells, similar to primary macrophages but unlike RAW264.7 cells, experience cell death following transfection with plasmid DNA, whereas they do not when transfected with mRNA (Van De Parre et al., 2005).

Validating results from macrophage-like cell lines with studies on primary macrophages is important for characterizing macrophage functions. Moreover, conducting animal experiments could further elucidate the *in vivo* relevance compared with the *in vitro* results of a single cell type.

#### 5.1.1 RAW264.7

The macrophage-like cell line RAW264.7 (Raschke et al., 1978) was isolated from BALB/c mice injected with the Abelson murine leukemia virus (A-MuLV), a replication-impaired virus carrying the v-abl tyrosine kinase oncogene (Hartley et al., 2008). When combined with a suitable type C helper virus, A-MuLV can transform embryo fibroblasts *in vitro* and trigger swift B cell lymphoid leukemia development *in vivo* (Twardzik et al., 1982). RAW264.7 cells are therefore an immortalized cancer cell line with macrophage-like capabilities (Taciak et al., 2018).

A-MuLV is a biosafety level 2 (BSL-2) agent (Feroz et al., 2021) posing a “moderate hazard” to lab personnel and the public, requiring specific safety protocols (Ta et al., 2019). Although macrophage cell lines originated from A-MuLV-induced tumors, it’s unclear if the A-MuLV genome contributed to cell transformation (Raschke et al., 1978). A-MuLV preparations often include a helper virus, but tests for replication-competent virus were negative when initially described (Raschke et al., 1978). RAW264.7 cells, as currently provided by the American Type Culture Collection (ATCC), express both ecotropic MuLV, which exhibits the biological characteristics of the Moloney isolate, and polytropic MuLV (Hartley et al., 2008). Polytropic viruses have a broad host range, allowing them to infect multiple species or various types of cell culture lines (Stoye and Coffin, 1987). The ecotropic virus, on the other hand, is characterized by its integration into cellular DNA and cell surface expression of A-MuLV antigens. Ecotropic refers to a virus with a limited host range, capable of infecting only one or a small number of species or cell culture lines. These findings, warrant caution in experimental design and data interpretation when using RAW264.7 cells (Hartley et al., 2008).

The RAW264.7 cell line is widely used to characterize macrophage phagocytosis (Kassas et al., 2012). The biggest advantage of RAW264.7 cells is that they can be transfected using various methods (electroporation, lipofection) and that they are also relatively easy to use for CRISPR-Cas9 lentiviral dropout screens. This facilitates mechanistic genetic unbiased screening studies (Smale, 2010; Cheung et al., 2015; Napier and Monack, 2017; Scinicariello et al., 2023). However, continuous passaging might even increase the accumulation of mutations and separate the cell line further from the definition of a primary macrophage (Stacey et al., 1993; Thompson et al., 1999; Cemma et al., 2015; Cheung et al., 2015; Paludan et al., 2019). Therefore, although this cell line serves as a practical tool for the initial screening of potentially important factors and functions in macrophages, it is important to consider continued passages of this cell lines could lead to gene depletion and compromise the immune functions of macrophages compared with primary macrophages and *in vivo* studies.

#### 5.1.2 J774

Similar to the RAW264.7 cell line (Raschke et al., 1978) the macrophage-like cell line J774 originally was discovered during cancer research (Ralph and Nakoinz, 1975). The J774 cell line is derived through re-cloning from the primary ascites and solid tumor J774.1 cell line. J774.1 cells were firstly described as cells derived from a murine reticulum sarcoma that exhibited macrophage-like morphology, presence of immunoglobulin receptors, phagocytic capacity and antibody mediated lysis of target cells (Ralph and Nakoinz, 1975). Notably, these original studies never claimed to have isolated macrophages but only described cells that display some typical macrophage properties (Ralph and Nakoinz, 1975; 1977). Nevertheless, similar to the RAW264.7 cell line, J774 cells were further characterized in terms of motility, phagocytosis, and antibacterial activity (Portnoy et al., 1988; Kant et al., 2002; Lam et al., 2009; Kresinsky et al., 2016). Notably, J774 cells, unlike RAW264.7 cells but similar to primary macrophages, undergo cell death after transfection with plasmid DNA, but not with mRNA (Van De Parre et al., 2005).

### 5.2 Primary mouse macrophages

The cultivation and utilization of primary macrophages offer several advantages over long-term cultured immortalized cell lines (Kaur and Dufour, 2012). They have a shorter lifespan than immortalized cell lines, leading to less accumulation of genetic mutations owing to long-term cultivation (He et al., 2023). Moreover, the origin of the primary macrophages may influence the results (Sreejit et al., 2020). It is of utmost importance to understand which primary macrophages are used in *in vitro* studies, as they differ due to differences in cultivation protocols and cultivation conditions.

#### 5.2.1 Peritoneal macrophages

Peritoneal macrophages (PMs) are a common *in vitro* model used to investigate tissue-resident macrophage response (Liu et al., 2018). Analysis of peritoneal macrophages provides insights into general macrophage biology and *ex vivo* behavior in response to various stimuli and disease models (Cassado Ados et al., 2015).

While working with the PMs, the isolation protocol should be considered. PMs can be obtained as unstimulated resident cells. The cells are isolated through peritoneal lavage (Ray and Dittel, 2010) and represent one of the largest sources of naïve, resident tissue macrophages (Yang et al., 2012b; Cassado Ados et al., 2015; Davies and Taylor, 2015; Davies et al., 2017; Gluschko et al., 2018). However, sorting with a macrophage-specific marker of choice (e.g., CD11b or F4/80) is recommended as the peritoneal lavage contains all cells present in the peritoneal cavity, mainly B cells and to a very small percentage also monocytes and neutrophils (Ray and Dittel, 2010; Gluschko et al., 2018). Relying solely on culture well adherence as the “sorting” step for macrophage purity may not be sufficient to achieve a completely pure peritoneal macrophage culture (Ponte-Sucre et al., 2007; Johnston et al., 2017), which is why authors suggest using the term peritoneal exudate cells for cells that have not been further purified (Johnston et al., 2017).

In this context, however, it must also be mentioned that a further flow cytometric analysis can influence the macrophage biology. Although a higher purity is achieved through purification, this in turn comes at the cost of influencing the biology of the cells. Moreover, extracting naïve peritoneal macrophages from mice has a significant drawback, as only a limited number of cells (1x10^6^ cells per mouse) are obtained, and only around 40% - 50% are macrophages (Ray and Dittel, 2010; Rios et al., 2017). Therefore, a greater number of mice must be sacrificed to obtain an adequate number of cells for experimental procedures (Zhang et al., 2008).

Consequently, to augment macrophage production, a sterile eliciting agent (such as thioglycollate) can be administered into the peritoneal cavity prior to cell collection (Misharin et al., 2012; Layoun et al., 2015). Thioglycollate consists of an infusion from beef, proteose peptone, sodium chloride, dextrose, sodium thioglycolate, bacto agar, methylene blue (Brewer, 1940; Schleicher and Bogdan, 2009). Composition of thioglycollate was shown to determine killing capacity of intracellular pathogens compared to naïve PMs. Reduction of agar and methylene blue in the eliciting agent increased killing capacity (Leijh et al., 1984).

Following thioglycollate administration, the macrophage yield per mouse increased by 10-fold (Layoun et al., 2015). Research has indicated that Brewer’s thioglycollate elicits significant recruitment of macrophages and leads to increased levels of activation compared to naïve PMs (Pavlou et al., 2017). Despite the increased macrophage yield, Brewer’s thioglycollate medium serves as an irritant, triggering an inflammatory response that leads to a swift decrease in overall resident monocyte/macrophage counts. Subsequently, there is an increase of peripheral inflammatory monocytes/macrophages, accompanied by a gradual restoration of resident macrophages (Davies et al., 2011, 2013b). This recruitment may or may not affect gene expression (Layoun et al., 2015). Furthermore, they exhibited an increase in lysosomal enzyme activity (Dy et al., 1978) and in phagocytic uptake (Pavlou et al., 2017).

Two studies have identified multiple leukocyte populations present in both the naïve and thioglycollate-elicited peritoneal cavities (Schleicher et al., 2005; Ghosn et al., 2010). However, maintaining high purity of macrophage culture is crucial for *in vitro* experiments. While freshly isolated thioglycollate-elicited peritoneal cells reportedly contain a high percentage of macrophages (86–95%) (Vodovotz et al., 1993; Schindler et al., 2001), their purity increases to nearly 99% through adherence (Zhang et al., 2008; Schleicher and Bogdan, 2009). It is worth noting that assessing macrophage percentage solely based on antigens like CD11b or F4/80, previously considered “macrophage specific”, might be misleading. This is because other myeloid cells such as neutrophils, eosinophils, and dendritic cells also express these antigens. Including markers against dendritic cells (CD11c), eosinophils (Siglec-F) and neutrophils (Ly6G) (Zhang et al., 2004; Connelly et al., 2022; Pfeifhofer-Obermair et al., 2022) might be useful because even minor levels of contaminating cells could impact the results of *in vitro* assays, leading to data misinterpretation (Schleicher et al., 2005; Martinez-Pomares and Gordon, 2008).

A separate study revealed that the proportion of macrophages from the peritoneal cavity was lower than previously reported (Schleicher et al., 2005; Ghosn et al., 2010), with a significant presence of eosinophils (Misharin et al., 2012). Furthermore, there are reports on eosinophil contamination in adherent cultures of peritoneal macrophages (Ruiz-Alcaraz et al., 2020). The contaminating cells affect the functional readouts of standard assays performed on macrophages (Tateyama et al., 2019). Therefore, eosinophils can hinder accurate interpretation of findings from *in vitro* studies using cultured thioglycollate-elicited peritoneal macrophages (Misharin et al., 2012; Tateyama et al., 2019). Eosinophils exhibit several cell surface markers, such as CD45 and CD11b, which are commonly found on other hematopoietic cells typically present in inflammatory sites, such as alveolar macrophages and neutrophils (Stevens et al., 2007). Siglec-F, a member of the sialic acid binding immunoglobulin-like lectin (Siglec) family, has been identified on murine eosinophils’ surface (Stevens et al., 2007). Siglec-F is predominantly expressed solely on eosinophils in blood and their precursors in the bone marrow of mice (Zhang et al., 2004). In addition to eosinophils, contamination of natural killer cells from peritoneal exudate cells can markedly influence conclusions regarding macrophage regulatory circuits (Misharin et al., 2012). To exclude the possible influence of NK cells in analyses, peritoneal macrophages, for example, can be induced in recombination activating gene 2 (RAG2)-gamma chain knock-out mice (Schleicher et al., 2005). To minimize the influence of eosinophils, double GATA-site (ΔdblGATA) knock-out mice could be used, for example, in which the development of eosinophils is severely impaired (Yu et al., 2002).

Another important point to consider is the changes in the metabolic activity of macrophages due to differences in the isolation protocols. Resident peritoneal macrophages in their naïve state exhibited lower metabolic activity than the elicited macrophages (Pavlou et al., 2017). Elicited macrophages display elevated levels of glycolysis and oxidative phosphorylation, potentially correlated with their enhanced phagocytic capacity and heightened maturation and activation levels (Pavlou et al., 2017). Gaining deeper insights into the molecular connections between metabolic pathways and cellular function is essential for devising strategies to regulate macrophage function through metabolic reprogramming.

#### 5.2.2 Bone marrow-derived macrophages

Compared to PM, BMDM are not a model for tissue-resident macrophages, as they are differentiated *in vitro* (Fejer et al., 2015). They are generated by flushing myeloid progenitor cells from the bone marrow of the hind legs of mice and stimulating with either recombinant M-CSF or L-929-cell-conditioned media (L929, source of M-CSF) to gain differentiated macrophages. M-CSF induces proliferation and differentiation of the progenitor cells into BMDM via M-CSF receptor-mediated signaling (Becker et al., 1987; Brugger et al., 1991; Klappacher et al., 2002; Stanley and Chitu, 2014; Ushach and Zlotnik, 2016).

The main advantage of using BMDM as a macrophage model is the amount of cells that are generated after isolation and differentiation (4-6 x 10^6^ cells per mouse) (Brigo et al., 2022). Differences in culture conditions (medium type, amount of M-CSF, and L929 usage) led to high variability in BMDM generation. However, clear description of the methodology is necessary for a high reproducibility of the results (Murray et al., 2014). In numerous research facilities, the L929 supernatant is favored over the use of recombinant M-CSF due to its cost-effectiveness and ability to produce significantly greater quantities of differentiated macrophages, as fewer animals need to be euthanized (Boltz-Nitulescu et al., 1987; Heap et al., 2021).

Before the availability of recombinant M-CSF, the traditional method, which remains prevalent in many laboratories (Yang et al., 2012b; Bringmann et al., 2013; Johnston et al., 2017; Koster et al., 2017; Nazir et al., 2017; Abuaita et al., 2018; Javmen et al., 2018; McTiernan et al., 2020), involved differentiating BMDM using the supernatant of the L929 immortalized fibroblast cell line, in which M-CSF and a variety of other factors are provided to the developing BMDM precursor (Tomida et al., 1984; Burgess et al., 1985; Portnoy et al., 1988; Pang et al., 2001). While certain strains of mouse L929 cells have the capability to generate significant levels of M-CSF (Rice et al., 2020), the composition of L929 supernatant can exhibit variability between batches, potentially leading to inconsistencies in experimental results (Warren and Vogel, 1985).

Despite exposure to other substances in the L929 supernatant (Trouplin et al., 2013), L929-derived macrophages exhibit similar phagocytic and pathogen eliminating abilities to M-CSF-derived macrophages (Rice et al., 2020). However, L929-derived macrophages showed distinct cytokine secretion patterns, with lower inflammatory cytokine levels and higher IL-10 secretion (de Brito Monteiro et al., 2020; Heap et al., 2021). They also display heightened metabolic activity and increased accumulation of dysfunctional mitochondria compared with M-CSF-derived macrophages. Although differences in metabolism and cytokine secretion exist, both types of macrophages demonstrate comparable microbicidal effectiveness (de Brito Monteiro et al., 2020).

Using mass spectrometry, the examination of L929 supernatant revealed 2,193 proteins, including notable amounts of M-CSF and other immune-regulating proteins like migration inhibitory factor (MIF), osteopontin, and chemokines such as CC-chemokine ligand (CCL) 2 and CCL 7. When differentiated with L929, macrophages showed a more robust anti-inflammatory M2-like phenotype compared to differentiation with M-CSF. Moreover, macrophages grown in L929 supernatant exhibited reduced responses to oxidative stress, as well as decreased activity in cell division and mitotic machinery (Heap et al., 2021).

In summary, these findings indicate that the choice of differentiation agent influences BMDM phenotypes and proteomes, leading to variations in biological functions. Consequently, it is crucial to recognize the biological implications of different BMDM differentiation methods and the resulting *in vitro* outcomes. Conversely, employing defined concentrations of M-CSF may alleviate experimental variability and improve the standardization of laboratory methodologies (Murray et al., 2014).

### 5.3 Cell line generation from mouse bone marrow-derived macrophages

Several methods for immortalizing mouse BMDM for generation of macrophage cell lines have been described; however, the low rates of transduction and transfection observed in macrophages pose challenges to these procedures (Zhang et al., 2009; Alamuru-Yellapragada et al., 2017; Warwick and Usachev, 2017; Poltavets et al., 2020). Some potential approaches for immortalizing primary BMDM are outlined in the next section of this review. However, one must carefully consider the advantages and disadvantages of these methods.

Genetic modulation of primary macrophages can lead to changes in their phenotype compared to the initial cell population (Xie et al., 2024). Nevertheless, immortalizing BMDM can lead to a reduction in animal usage (Spera et al., 2021). BMDM from genetically modified mice are frequently employed to analyze mechanisms of the immune system and having a reservoir of these immortalized BMDM readily available facilities the conduction of *in vitro* studies and reduces reliance on live animals (De Nardo et al., 2018; Spera et al., 2021).

#### 5.3.1 Overexpressing oncogenes or a transcription factor in bone marrow-derived macrophages

A method for immortalizing macrophage populations from particular mouse strains by utilizing the Cre-J2 retroviral infection technique (De Nardo et al., 2018). This method of immortalization involves infecting cells with the J2 recombinant retrovirus, which is derived from a replication-defective 3611-Moloney sarcoma virus (MSV) and contains the v-raf and v-myc oncogenes (Spera et al., 2021).

The J2 virus itself lacks the essential viral packaging proteins (gag-pol, env), rendering it replication-deficient. Therefore, a viral packaging helper cell line called Psi-Cre-J2 (derived from NIH 3T3 fibroblasts) is utilized to generate recombinant Cre-J2 retrovirus. The Cre-J2 retroviral infection technique for immortalization has proven effective on various murine macrophage populations, such as those derived from bone marrow, fetal liver, spleen, and microglia (Blasi et al., 1985; Roberson and Walker, 1988; Cox et al., 1989; Blasi et al., 1990).

A commercially available immortalized murine macrophage cell line, designated as BM A3.1A7, originates from adherent macrophages extracted from the bone marrow of adult female C57BL/6 mice. These cells have been rendered immortal by the introduction of elevated levels of raf and myc oncogenes (Kovacsovics-Bankowski and Rock, 1994).

Currently only one study analyzed the immortalized murine macrophage cell line, BM A3.1A7 for their macrophage capabilities. They demonstrated that BM A3.1A7 polarize into either M1-like macrophages, identified by their secretion of inflammatory cytokines such as IL-1β, IL-6, IL-12, and tumor necrosis factor (TNF), along with increased expression of inducible nitric oxide (NO) synthase (iNOS), or M2-like macrophages characterized by their typical elevated ARG1 activity (Banete et al., 2015). Further studies and comparisons with other primary macrophages or other relevant models are needed to better assess the value of the model.

Moreover, hematopoietic precursors are immortalized by retroviral transduction with an estrogen-inducible form of the transcription factor homeobox B8 (Hoxb8) (Wang et al., 2006). Hoxb8 promotes self-renewal and halts differentiation (Leithner et al., 2018). In the presence of elevated levels of the hormone β-estradiol that exceed the physiological range, Hoxb8 is transcriptionally active. By removing β-estradiol, Hoxb8 is inactivated, leading to differentiation of the immortalized progenitor cells depending on the applied cytokine cocktail for the desired myeloid subset (Wang et al., 2006; Odegaard et al., 2007). Therefore, these cells can be grown in cell culture for weeks, providing them with sufficient time to be genetically modified, while still remaining capable of maturing into DCs, macrophages, or granulocytes (Redecke et al., 2013). Macrophages derived from the Hoxb8 lineages exhibited comparable phenotypic and functional attributes and similarly elevated the expression of activation-related genes when stimulated with LPS, as observed in primary macrophages (Wang et al., 2006; Odegaard et al., 2007).

Advantages of these cells are that they can be readily produced from the bone marrow or fetal liver of any mouse strain (Hammerschmidt et al., 2018). Additionally, Hoxb8 cells can be efficiently modified using viral transduction and CRISPR-mediated genome editing (Di Ceglie et al., 2017; Hammerschmidt et al., 2018). They are a convenient tool for protein overexpression or knockdown experiments (Bromberger et al., 2022). One disadvantage of these cells is the heterogeneity, as the initial bone marrow population can be diverse, potentially impacting the uniformity of the resulting cells (Yu and Scadden, 2016).

#### 5.3.2 Cas9+-immortalized macrophages

Genetic manipulation of macrophages is in general cumbersome (Zhang et al., 2009). There are for instance protocols for RNAi delivery into macrophages (Siegert et al., 2014) or retroviral transduction of bone marrow progenitors (De Veerman et al., 1999). The discovery of the RNA-guided endonuclease Cas9 and its use as gene scissors (‘genome editing’) has opened new technical possibilities to search genome-wide with CRISPR (clustered regularly interspaced short palindromic repeats)-Cas9 for regulators of biological processes starting from the phenotype with so-called ‘forward genetic screens’ (Joung et al., 2017). Introducing Cas9 protein into cells poses a difficulty within the CRISPR-Cas9 process (Liang et al., 2015). To overcome this challenge Cas9 knock-in mice facilitate the production of various knock-outs in immortalised cells, which was already shown in immortalised DCs of Cas9 expressing mice (Parnas et al., 2015).

As previously mentioned, hematopoietic precursors immortalized by retroviral transduction with Hoxb8 can be cultured for weeks, allowing genetic modifications and maturation into DCs, macrophages, or granulocytes (Wang et al., 2006; Redecke et al., 2013), as Hoxb8 expression facilitates self-renewal and halts differentiation (Leithner et al., 2018). To overcome the inducibility of the Hoxb8 overexpression via removal of β-estradiol, bone marrow of the Cas9-transgenic mice was immortalized by lentiviral transduction, introducing a doxycycline-regulated version of the transcription factor Hoxb8. These cells can be cultured consistently for weeks when doxycycline and puromycin are added. Moreover, these cells facilitate CRISPR/Cas9 technology, as the Cas9 gene is expressed in the cells (Hammerschmidt et al., 2018).

A recent study focused on the notable differences and constraints observed between primary macrophages and immortalized cell lines by creating a new immortalized macrophage cell lines using the CRISP/Cas9 technique (Roberts et al., 2019). Roberts and colleagues introduced ER-HoxB8 by retroviral transduction into hematopoietic stem cells from Cas9 expressing mice, and performed a comprehensive comparison between Cas9+-immortalized macrophages (CIMs) and BMDM, the primary macrophage type. Through a series of meticulously designed experiments, they examined various macrophage functions, including iNOS expression, NO generation, bacterial phagocytosis, and antibacterial activity. CIMs were similar to BMDM, the only significant difference was that killing of *Listeria monocytogenes* was enhanced, which they hypothesize is a result of improved early elimination through the phago-lysosomal pathway. The growth rate of *M. tuberculosis* was similar in CIMs to that observed in BMDM. CIMs and BMMs showed increased expression of iNOS and produced comparable levels of NO when stimulated with the combination of LPS and interferon-γ (Roberts et al., 2019).

## 6 Immortalized versus primary mouse macrophages: advantages and disadvantages of *in vitro* models

As previously mentioned, numerous studies concentrate exclusively on the primary role of macrophages, which is phagocytosis. Nevertheless, macrophages are not merely scavengers; they respond to diverse environmental cues by generating complex reactions, such as generating reactive oxygen and nitrogen species (ROS and RNS) as well as pro- or anti-inflammatory cytokines and chemokines, which depend on their polarization (Arango Duque and Descoteaux, 2014; Mosser et al., 2021).

It is becoming evident that macrophage-like cell lines differ from primary macrophages, while primary macrophages vary based on isolation and cultivation methods. The subsequent section of this review will illustrate these distinctions.

### 6.1 Differences between naïve PM and BMDM

Primary mouse macrophages, including BMDM and PM, are commonly employed *in vitro* to investigate various mechanisms such as infection control (Schatz et al., 2016; Brigo et al., 2021), phagocytosis (Herb et al., 2018; Gluschko et al., 2022), and cytokine production (Sasse et al., 2022; Brigo et al., 2023). Interestingly, these two macrophage types exhibit distinct functional differences.

Macrophages in various anatomical locations may exhibit diverse degrees of heterogeneity (Geissmann et al., 2010; Gautier et al., 2012; Gordon et al., 2014; Komohara et al., 2014; Hume et al., 2023). Employing flow cytometry, the observations revealed that BMDM formed tight clusters in both forward scatter and side scatter data, contrasting with naïve PMs, which appeared dispersed among at least two distinct populations (Zajd et al., 2020). These findings suggest that PMs display notable heterogeneity. Conversely, BMDM exhibited a higher degree of uniformity, likely attributable to their induction by M-CSF (Zhao et al., 2017). Moreover, PMs exhibit larger cell sizes compared to BMDM due to containing more cytoplasm and increased lysosomal content (Wang et al., 2013). In addition, proliferation is enhanced in BMDM, cell number increased from day 4 and continued to rise until day 14, reaching a 60-fold increase over baseline compared to PMs, which showed no proliferation during the 14-day culture period (Wang et al., 2013).

BMDM, unlike PM, fail to activate an antimicrobial phagocytosis variant known as LC3-associated Phagocytosis (LAP) (Heckmann and Green, 2019; Herb et al., 2020). Not only do BMDM fail to induce LAP during bacterial infection, but they also exhibit significantly reduced capacities in phagosomal ROS production and intracellular bacteria killing (Gluschko et al., 2022). ROS production via the NADPH oxidase (Nox) 2 is crucial for LAP induction (Martinez et al., 2015; Koster et al., 2017; Gluschko et al., 2018; Herb et al., 2020). Our investigations have revealed markedly lower protein levels of several Nox2 subunits in BMDM compared to PM, elucidating the pronounced reduction in ROS production in BMDM (Gluschko et al., 2022). Additionally, upstream components essential for Nox2-derived ROS production, namely the integrin Mac-1 and the sphingomyelinase ASMase, are also significantly reduced on the protein level in BMDM compared to PM (Gluschko et al., 2018).

Furthermore, time of cytokine secretion was slower in BMDM (24 hours post-infection) compared to PM (5 hours post-infection) (Herb et al., 2019). Bisgaard et al. conducted a comparative study on the behavior of PM and BMDM in an *in vitro* atherosclerosis model, revealing dramatic differences in chemokine and cytokine expression following cholesterol treatment, with PM exhibiting a tendency towards an M1-like phenotype and BMDM towards an M2-like phenotype (Bisgaard et al., 2016). Wang et al. showed that BMDM exhibited the highest phagocytic ability compared to PMs (Wang et al., 2013). Interestingly, phagocytosis efficiency diminishes in PMs of old mice compared to the younger counterpart. On the other hand, there were no discernible age-related impairments in phagocytosis observed in BMDM, indicating the absence of intrinsic defects within these cell populations (Linehan et al., 2014).

### 6.2 Differences between elicited PM and BMDM

Several studies have investigated the differences between thioglycollate-elicited PM (TG-PM) and BMDM. Weber and Schilling demonstrated that lysosomes isolated from TG-PM exhibited enhanced lysosomal numbers, more active cathepsin B, and higher levels of pro-cathepsin D and Lysosomal-associated membrane protein 1 (LAMP1) compared to lysosomes from BMDM (Weber and Schilling, 2014). In comparison to the study of Bisgaard et al. (Bisgaard et al., 2016) a recent study by Zajd and colleagues revealed that TG-PM displayed M2 surface markers, while BMDM exhibited M1 surface markers (Zajd et al., 2020). Furthermore, TG-PM showed reduced responsiveness, including decreased phagocytic capacity, weaker response to additional polarization stimuli, and diminished secretion of cytokines and chemokines (Zajd et al., 2020). However, both studies miss a comparison of TG-PM and BMDM to non-elicited naïve PM. This comparison is crucial as the injection of thioglycollate induces sterile inflammation, significantly altering naïve peritoneal macrophages before isolation and the start of experiments (Weber and Schilling, 2014; Zajd et al., 2020).

The study by Zajd et al. (Zajd et al., 2020) showed that both types are morphologically similar in flow cytometry. BMDM, however, exhibit higher phagocytic activity and upregulate chemokine and cytokine expression more robustly in response to polarization. This heightened responsiveness of BMDM reflects intrinsic differences, while TG-PM responses are influenced by their differentiation within the whole animal context. BMDM express higher levels of inflammatory markers such as Ly6C and CD64 and show increased expression of pattern recognition receptors like TLR2 and TLR4 compared to TG-PM. However, their modest expression of MHCII suggests M1-like skewing rather than full activation (Zajd et al., 2020).

Despite the observed differences, these studies (Weber and Schilling, 2014; Bisgaard et al., 2016; Gluschko et al., 2018; Herb et al., 2019; Zajd et al., 2020) suggest that naïve peritoneal macrophages are the preferred tissue-resident model due to their more natural state (Chen et al., 2021c). However, BMDM differentiated with M-CSF demonstrate enhanced sensitivity to polarizing cytokines and improved phagocytic abilities compared to PMs. Consequently, they serve as a favorable model for investigating macrophage plasticity and immune responses to infections (Zajd et al., 2020).

They represent a valuable alternative, particularly in situations where access to a large pool of animals for isolation purposes is limited (Zhang et al., 2008; Layoun et al., 2015). However, the use of TG-PM, with their highly altered phenotype and reduced responsiveness, warrants caution, even though obtaining higher cell numbers for experimental procedures is very tempting.

### 6.3 Differences between PM and immortalized macrophage cell lines

Comparison of J774 to PM revealed that PM store substantial amounts of cholesterol ester from unmodified low-density lipoprotein (LDL) in contrast to J774 cells (Tabas and Boykow, 1987). Based on these findings, J774 macrophages exhibit a significantly more active acyl-coenzyme A (CoA):cholesterol acyltransferases (ACAT) cholesterol esterification pathway in the presence of LDL compared to primary PM. Additionally, there’s a notable distinction in the stimulation of ACAT between acetyl-LDL and LDL in mouse PM, even when the lipoproteins are matched for degradation (Tabas et al., 1987).

Infection control to *Cryptococcus neoformans* var. *grubii* strain H99 (serotype A) was different in J774 compared to primary PMs. The J774 macrophage-like cell line demonstrated a tendency to activate caspase-1 and caspase-3 throughout the infection process. Subsequently, primary PMs were examined, revealing activation of caspase-3. Despite increased ROS production, fungal control was diminished in PM compared to J774 cells (Coelho et al., 2015).

Moreover, PMs displayed higher basal NF-κB levels compared to RAW264.7 cells and exhibited faster NF-κB nuclear translocation kinetics upon low-dose LPS activation. Notably, primary mouse PM showed particularly rapid NF-κB translocation kinetics compared to immortalized cell lines (Bagaev et al., 2019).

### 6.4 Differences between BMDM and immortalized macrophage cell lines

Differences between primary macrophage types primarily arise from variations in isolation and cultivation protocols (Murray et al., 2014). However, macrophage-like cell lines exhibit significant discrepancies compared to primary macrophages due to their cancerous origin (Ralph and Nakoinz, 1975; Raschke et al., 1978; Tsuchiya et al., 1980).

While some reports found no differences between RAW264.7 cells and BMDM in basic parameters such as antibacterial and antiparasitic activity (Cemma et al., 2015; Frank et al., 2015), other studies highlighted remarkable distinctions. For instance, a study investigating the regulatory role of FAS-associated factor 1 (FAF1) on ROS production (Kim et al., 2019) showed that RAW cells produced significantly less IL-6 and IL-12 but more NO compared to BMDM after infection with *Listeria monocytogenes* (*L.m*). Total cellular ROS production was equal in both cell types (Kim et al., 2019).

Huang et al. detected higher levels of septins in BMDM compared to RAW cells, indicating their importance in phagosome formation (Huang et al., 2008). Furthermore, in another study, a comprehensive proteomics analysis of phagosomes from RAW264.7 and BMDM was conducted (Guo et al., 2015). Over 2,500 phagosomal proteins were quantified, and significant differences in important receptors like mannose receptor 1 and Siglec-1 were analyzed. Additionally, Guo et al. observed that phagosomes in BMDM undergo more rapid maturation through fusion with endosomes and lysosomes, a phenomenon confirmed through fluorogenic phagocytic assays, compared to the immortalized macrophage cell line (Guo et al., 2015). Analysis of the phagosomal proteome of both cell types revealed that 58 proteins were unique for BMDM and 17 were unique for RAW phagosomes, with immune-relevant proteins (TLR3, TLR9, complement receptors, mannose receptor 1 and several integrins and galectins) more abundant in BMDM phagosomes (Guo et al., 2015). This discrepancy between RAW cells and tissue macrophages becomes more evident considering the decreased integrin protein levels in BMDM compared to peritoneal macrophages (Gluschko et al., 2018).

Interestingly, BMDM and RAW cells exhibited comparable phenotypes, characterized by elevated levels of CD11b and F4/80 expression (Berghaus et al., 2010). However, although both populations shared similarities, their phenotypes were not identical; RAW cells displayed notably higher CD14 expression emphasizing their distinct characteristics. Furthermore, both BMDM and RAW cells demonstrated similar responses to TLR 3 stimulation with poly I:C, indicating their shared activation pathway in the monocyte-macrophage differentiation process. This similarity in responsiveness to various stimuli suggests a common functional point in their differentiation (Berghaus et al., 2010).

Additionally, J774 cells showed no antilisterial activity in comparison to BMDM (Portnoy et al., 1988). Andreu et al. observed an increased infection control to *Mycobacterium tuberculosis* in BMDM compared to J774 cells (Andreu et al., 2017). Moreover, infection control with BMDM is faster and stronger (Andreu et al., 2017). The J774 macrophage-like cell line tended to activate caspase-1 and caspase-3 during *C. neoformans* infection, whereas BMDM activated caspase-1, -3, and -8. Protein expression analysis showed upregulation of receptor-interacting protein (RIP) and apoptosis-inducing factor (AIF) in J774 cells early in infection, while BMDM activated AIF and released cytochrome c (Coelho et al., 2015). Additionally, J774 cells displayed increased LDH levels, indicating necrotic cell death, unlike BMDM, which exhibited classical apoptotic features (Coelho et al., 2015).

## 7 Additional variables that may impact the functionality of macrophages

The origin of macrophages (Gordon et al., 2014; Komohara et al., 2014), duration of culture (Chamberlain et al., 2015), characteristics of biomaterial surfaces (Jones et al., 2007), culture media (Kawakami et al., 2016, 2017) and addition of supplements to the culture conditions (Rinsky et al., 2017; Antonsen et al., 2023) collectively influence the phenotype of macrophages in culture.

### 7.1 Effects of fetal bovine serum and cell culture media on macrophage culture

Addition of fetal bovine serum (FBS) or fetal calf serum (FCS) to the cell culture medium serves as a vital supplement leading to facilitated cell growth and proliferation (Karnieli et al., 2017). Since the 1950 addition of FBS/FCS into cell culture media is a standard procedure (Puck et al., 1958). FBS consists out of crucial elements necessary for cell growth and upkeep, such as hormones, vitamins, transport proteins, trace elements, and growth factors (Fang et al., 2017). FBS, which is a by-product of cattle husbandry, is acquired from the blood of a bovine fetus during the slaughtering of a pregnant cow (Fang et al., 2017). Although various serum-free medium formulations exist for mammalian and insect cell lines as well as primary cultures, transitioning to serum-free media requires extensive literature review and manufacturer searches for suitable formulations (Brunner et al., 2010; Fang et al., 2017).

FBS is a complex and variable mixture that can contain contaminants and varies significantly due to geographical, seasonal, and environmental factors, contributing to lot to lot differences (Fang et al., 2017).

In a study, various commercially available FBS were analyzed for their effect on epithelial cells. The findings revealed that various FBS samples markedly stimulated IL-8 secretion in the cells, while they did not elicit secretion of TNF and IL-1β. Conversely, some FBS samples had no impact on the secretion of IL-8, TNF, and IL-1β (Liu et al., 2023b).

Moreover, exosomes found in FBS, influenced primary macrophages from Fisher 344 rats, when cultured with LPS. The macrophages demonstrate a dose-dependent decrease in IL-1 compared to macrophages cultured in medium supplemented with exosome-depleted FBS. Furthermore, the inclusion of fetal bovine exosomes also led to reductions in macrophage TNF-α and IL-6 levels (Beninson and Fleshner, 2015).

As exosomes have the potential of being a reliable biomarker as they are stable in body fluids, reflect the physiological state of their parent cells, and facilitate intercellular communication through the transfer of biomolecules (Zhou et al., 2020; Mathew et al., 2021). The high levels of serum proteins, including exosomes derived from bovine cells, can potentially contaminate exosome samples, leading to significant impurities and artifacts in the yields (Biadglegne et al., 2021). Therefore, a serum free approach is necessary. Abramowicz et al. showed that the presence of high levels of serum proteins contaminating exosomes can cause significant issues in the harvest, isolation, and processing of exosomes (Abramowicz et al., 2018).

Another interesting point to consider is hemolysis. Hemolysis can greatly affect FBS production by releasing free hemoglobin and other intracellular contents into the serum, which can alter its composition and impact its performance and consistency in cell culture applications (Bowen et al., 2010; Arigony et al., 2013; Shah et al., 2016; Chelladurai et al., 2021).

Moreover, trace amounts of endotoxin (lipopolysaccharide: LPS) are believed to contaminate commercially available FBS (Kirikae et al., 1997). Beninson et al. described that endotoxin contamination affects cultured cells by inducing the production of various active mediators, such as TNF, leading to diverse cellular responses (Kirikae et al., 1997). Interestingly, tolerance to endotoxins is a well-known characteristic of macrophages, resulting in a modified macrophage response (Butcher et al., 2018). The innate immune response to infection or injury is shifted from a pro-inflammatory to an anti-inflammatory (Vergadi et al., 2018). Following an initial exposure to LPS in monocytes/macrophages a temporary state of “endotoxin tolerance”, characterized by reduced responsiveness to LPS, is observed (Rajaiah et al., 2013).

In animal models, endotoxin tolerance has two phases: an early phase with altered cellular activation and a late phase involving the development of specific antibodies against the polysaccharide side chain of Gram-negative organisms (West and Heagy, 2002). The physiological role of tolerance is to protect host tissue from damage caused by prolonged production of pro-inflammatory cytokines (Rogovskii, 2020). While mostly reversible, LPS tolerance creates a hybrid macrophage activation state that is primarily pro-inflammatory but includes distinct anti-inflammatory regulatory features (Butcher et al., 2018).

Not only FBS may be contaminated by endotoxins, but culture media are also a potential source. Currently, most commercially prepared media are tested for endotoxin and certified to contain less than 0.1 ng/mL. However, reagents added to the medium can also introduce endotoxins (Ryan, 2004). Dumoulin et al. tested five different batches of commercially prepared bovine serum albumin and found endotoxin levels ranging from 0.1 to 12 ng/mL (Dumoulin et al., 1991). Additionally, another study found that some media additives, such as erythropoietin, contained endotoxin levels as high as 50 ng/mL (Case-Gould, 1984). Thus, endotoxin testing is crucial concerning cell culture experiments to ensure the reliability and validity of experimental results (Nomura et al., 2017; Molenaar-de Backer et al., 2021).

Moreover, usage of different cell culture media can affect the phenotype of macrophages. Kawakami et al. presented novel evidence that J774 exhibits varied activated macrophage phenotypes in response to LPS and/or interferon-gamma (IFN-γ) stimulation when cultured in either Ham’s F-12 medium (F-12) or Dulbecco’s modified Eagle medium (DMEM). Specifically, the production of NO and certain cytokines was notably higher in DMEM compared to F-12 during macrophage activation (Kawakami et al., 2016).

### 7.2 Influence of biomaterial surfaces on macrophage culture

Macrophages can be distinguished from other cell types based on their capacity to efficiently adhere and proliferate on both glass and plastic surfaces, therefore, the surface of the culture dish is key (Fleit et al., 1984). Normal tissue cells typically do not survive when suspended in a fluid, thus they are considered to be anchorage dependent (Ruoslahti and Pierschbacher, 1987; Discher et al., 2005). These cells must adhere to a solid, which can range from rigid glass to a surface softer than baby skin. The way some cells behave on soft materials is crucial for identifying important phenotypes (Merten, 2015; Discher et al., 2017).

Epithelial cells and fibroblasts were the first to be reported as detecting and responding differently to soft versus stiff substrates (Pelham and Wang, 1997; Deroanne et al., 2001). Although the molecular pathways are still not fully understood, muscle cells, neurons, and various other tissue cells have since been shown to sense substrate stiffness (Wang et al., 2000; Engler et al., 2004). The increasingly clear and affirmative answer to whether cells perceive and respond differently to the rigidity of conventional materials compared to more compliant tissues, gels, or sublayers of cells is significant for its impact not only on standard cell culture but also on understanding disease processes, morphogenesis, and tissue-repair strategies (Discher et al., 2005; Guo et al., 2006; Majhy et al., 2021).

Macrophage cytokine expression is contingent upon both the cell type and the culture surface (Chamberlain et al., 2015). In a study by Chamberlain et al. with primary macrophages or immortalized macrophage cell lines, the cells exhibit a distinct response due to three different solid surfaces of the culture dish. Cell lines demonstrate variability among themselves in terms of adherent morphology, proliferation, cytokine expression, and cell surface marker expression (Chamberlain et al., 2015).

Conversely, another study indicated that the surface chemistry of these four non-cytotoxic biomaterials had only a modest impact on cytokine production (Schutte et al., 2009). Whereas divergences were noted in the capacity of cells to adhere to and subsequently proliferate on polymer surfaces in murine monocyte-macrophages (RAW264.7 and J774), murine macrophage (IC-21) and murine fibroblast (NIH 3T3) cell lines (Godek et al., 2004).

The material surface chemistry influences the phenotypic expression of macrophages. A study found that macrophages on different surfaces showed varying cytokine/chemokine profiles, with hydrophilic/neutral surfaces resulting in fewer but more highly activated cells. Over time, a shift from proinflammatory to anti-inflammatory cytokine production was observed, indicating a resolution of the inflammatory response (Jones et al., 2007).

Collectively, signaling alterations, as well as functional changes in macrophages may be dependent on the culture dish surface, serum addition and cell culture media.

## 8 Other macrophage models

### 8.1 Immortalized human macrophage cell lines

#### 8.1.1 THP-1

THP-1 cells were isolated from the peripheral blood of a one-year-old with acute monocyte leukemia (Tsuchiya et al., 1980). Similar to the RAW cell line, THP-1 monocyte-like cells are constantly proliferating and can accumulate several mutations during passaging (Noronha et al., 2020). Despite the fact, that these cells were originally mainly used for leukemia cancer research (Fabian et al., 2006; Yang et al., 2012a; Alves et al., 2018; Chernoryzh et al., 2019), they quickly adapted to a human monocyte/macrophage model cell line (Auwerx, 1991; Chanput et al., 2014; Bosshart and Heinzelmann, 2016; Zhang et al., 2019), which can be achieved by differentiation into macrophage-like cells with phorbol 12-myristate 13-acetate (PMA) (Koster et al., 2017; Sedlyarov et al., 2018) or human rM-CSF treatment (Muczynski et al., 2016). Furthermore, like BMDM, there is a lack of consistent differentiation protocol for THP-1 cells. In a study comparing conditions, researchers found that an optimal PMA concentration enables THP-1 cells to combat intracellular bacteria, while high concentrations lead to faster cell death. Lower concentrations support cell survival and effective defense against intracellular bacteria, like primary human macrophages (Aldo et al., 2013; Starr et al., 2018).

THP-1 cells offer several technical benefits compared to human primary monocytes or macrophages. One key advantage is their uniform genetic background, which reduces variability in cell phenotypes (Chanput et al., 2015). Like the RAW cell line, THP-1-derived macrophages can be easily transfected with plasmid DNA (Maess et al., 2011; Bosshart and Heinzelmann, 2016). Additionally, it is relatively straightforward to genetically modify THP-1 cells using small interfering RNAs (siRNAs) to downregulate specific protein expressions (Chanput et al., 2010).

Several publications have compared the responses of THP-1 monocytes with those of human PBMC-monocytes. The results are well summarized by Chanput et al. (Chanput et al., 2014). Differences have been observed in the levels of gene expression and cytokine secretion, as well as in the baseline gene expression (Chanput et al., 2014).

Thus, a limitation of using THP-1 cells is that their malignant background and cultivation under controlled conditions may result in different sensitivities and responses compared to PBMCs and human monocyte-derived macrophages (Schildberger et al., 2013; Hoppenbrouwers et al., 2022). For instance, compared to THP-1 cells, monocytes are significantly more responsive to LPS. This notable LPS responsiveness in human peripheral blood monocytes is primarily due to the high expression levels of CD14 (Bosshart and Heinzelmann, 2016). THP-1 cells express low levels of CD14, making them a poor model for studying LPS responses compared to primary monocytes (Bosshart and Heinzelmann, 2004). LPS concentrations that can trigger severe, life-threatening reactions in an *in vivo* system are non-toxic to THP-1 cells (Prajitha and Mohanan, 2021)

#### 8.1.2 BLaER1 cell line

The human B-cell precursor leukemia cell line BLaER1 was derived from the transfection of the Burkitt Lymphoma Cell Line Seraphina, an acute lymphoblastic leukemia (ALL) cell line with the CCAAT/enhancer-binding-protein (C/EBPα), the estrogen receptor (ER) coupled to green fluorescent protein (GFP) (Gaidt et al., 2018). Following transfection, cells were sorted based on GFP expression, resulting in the generation of a single subclone (Rapino et al., 2017). The progenitor cell line was derived from the bone marrow of a female patient presenting with a chromosomal translocation t(1;19), trisomy 8 and ALL (Jack et al., 1986). Through tamoxifen or β-estradiol, the transcription factor C/EBPα is activated leading to the conversion of immature/mature B-cells into macrophage-like cells (Xie et al., 2004; Bussmann et al., 2009). BLaER1 cells had a transcriptome that started in a position close to that of peripheral blood B-cells before induction of C/EBPα and ended close to that of normal macrophages after 3-4 days of C/EBPα activation (Bussmann et al., 2009; Rapino et al., 2013). Interestingly, the frequency of induced lymphoid cells converting into macrophages is significantly greater than that observed in the reprogramming of somatic cells into induced pluripotent stem cells by transcription factors associated with embryonic stem cells (Laiosa et al., 2006; Takahashi and Yamanaka, 2006; Bussmann et al., 2009). Infection experiments with *Escherichia coli* and *Candida albicans* showed that the reprogrammed macrophages function as phagocytic cells (Bussmann et al., 2009; Rapino et al., 2013). The capacity of BLaER1 monocytes to support *Leishmania* parasite infection and subsequent activation is comparable to that of primary human macrophages (Volkmar et al., 2024). Moreover, cytokine response due to infection is comparable to M-CSF-derived macrophages and GM-CSF-derived macrophages (Volkmar et al., 2024).

An advantage of the BlaER1 cells is that the genetic modification can be achieved in the undifferentiated B-cell form through the utilization of established CRISPR-Cas9-based methodologies, conferring a distinct advantage over the limited capacity for genetic manipulation observed in other monocytic cells (Gaidt et al., 2016; Schussler et al., 2023; Volkmar et al., 2024).

The selection of a human cell model is a crucial decision that requires careful consideration. For instance, alternative NLRP3 activation has only been documented in BlaER1 cells, highlighting the importance of choosing an appropriate model (Zito et al., 2020). This species-specific NLRP3 inflammasome pathway was identified in human and porcine peripheral blood mononuclear cells, yet was absent from those of murine origin and the THP-1 cell line (Gaidt et al., 2016, 2018).

### 8.2 Primary human macrophages

#### 8.2.1 Peripheral blood monocytes cells

Differentiated macrophages from human peripheral blood monocytes (PBMC) represent another type of commonly used human macrophage cells. In addition to routine blood collection, a technique known as apheresis can be employed for the automated isolation of specific blood components. When isolating PBMCs, this automated process is referred to as leukapheresis (Liu et al., 2015).

Protocols to differentiate PBMC into macrophages with human rM-CSF (Stanley et al., 1976; Becker et al., 1987; Brugger et al., 1991; Takahashi et al., 1996; Erbel et al., 2013) or GM-CSF (Herbst et al., 2020) were described, but studies highly differ in their technical approach to date (Mehta et al., 1996; Pilling et al., 2017; Sedlyarov et al., 2018; De et al., 2020; Herbst et al., 2020; Nielsen et al., 2020; Braga et al., 2021). Diverse isolation methods may significantly affect the macrophage outcome, resulting in variations in yield, purity, viability, and cellular phenotype (Nielsen et al., 2020). For instance, leukapheresis yields higher amounts of cells than routine blood collection (Liu et al., 2015).

Factors occurring before or during blood sampling can affect PBMCs and consequent the results of the assay. For instance, induction of stress has been associated with inhibiting cytokine synthesis and the release of immunosuppressive cytokines (Elenkov and Chrousos, 2002).

Another factor impacting PBMC function is nutritional status. This influence, whether due to acute starvation from long-term overall malnutrition or specific nutrient deficiencies, can diminish the organism’s immune response capacity to combat pathogens or respond to vaccination (Bourke et al., 2016).

Time of blood sampling is another factor. In humans and mice, the immune system, including lymphocyte movement between blood and tissues, follows circadian rhythms, leading to variations in immune cell counts throughout the day (Scheiermann et al., 2013). Moreover, before isolation, the time and temperature of blood shipping and storage can impact both the isolation process and subsequent assays. Besides reducing cell viability, granulocyte contamination is the primary cause of sample quality degradation over time (McKenna et al., 2009).

Thoughtful evaluation of monocyte isolation techniques is crucial when designing *in vitro* experiments involving PBMCs.

#### 8.2.2 Immortalized pluripotent stem cell-derived macrophages

Induced pluripotent stem cells (iPSCs) provide the opportunity to create various disease-relevant cell types from any genetic background through the processes of cellular reprogramming and directed differentiation (Yamanaka and Blau, 2010; Yanagimachi et al., 2013). iPSCs are undifferentiated pluripotent cells that have the potential to be cultivated for an unlimited period of time (Takahashi et al., 2007; Yamanaka, 2007).

The five-step monocytic lineage differentiation protocol was published by Yanagimachi et al., 2013. This protocol differentiates mature macrophages from human iPSCs through the monocyte stage. In the last step CD14+ monocytic lineage-cells are cultured with M-CSF for one week for macrophage differentiation (Yanagimachi et al., 2013).

The iPSC-derived macrophages showed a notable capacity for phagocytosis of bacteria, although it was somewhat diminished in comparison to blood monocyte-derived macrophages. However, it was observed that the pro-inflammatory responses and transcriptomic profiles were comparable to blood monocyte-derived macrophages (Monkley et al., 2020). It has been suggested that a bias towards a more anti-inflammatory phenotype, perhaps more similar to tissue resident macrophages, may be one reason for the lower phagocytic activity of iPSC-derived macrophages (Buchrieser et al., 2017; Haideri et al., 2017).

The procurement of patient-derived tissue-resident macrophages represents a significant challenge, largely due to their inherent genetic variability and the technical difficulties associated with genetically modifying them (Gastman et al., 2020; Yang et al., 2024). iPSC can be produced from a patient with a specific genetic background and modified by multiple mechanisms, such as lentiviral transduction or CRISPR-Cas9 gene editing (Yamanaka, 2007; Buchrieser et al., 2017; Chehelgerdi et al., 2023). Moreover, the possibility of genetically modifying the original iPSC culture line in order to generate various specific genetic variants in large numbers may represent a valuable approach for large-scale studies, such as those related to drug development (Shi et al., 2017; Huang et al., 2019).

### 8.3 Comparison of human and mouse macrophages

An area of contention in macrophage biology revolves around the perceived disparities between rodent and human macrophages (Wynn et al., 2013). It has been proposed that human macrophages may be fundamentally different from their rodent counterparts. Especially concerning the expression of ARG1 and iNOS as they play an important role in immune defense, as it is challenging to induce human monocyte-macrophage cell lines to produce iNOS readily (Murray and Wynn, 2011a, 2011b). Moreover, the antimicrobial product, itaconic acid, is two hundred times lower in primary human monocytes compared to mouse cells (Michelucci et al., 2013).

Additionally, microarray analysis comparing human monocyte subsets to mouse subsets revealed gene expression patterns that diverge between species (Ingersoll et al., 2010). A more recent study used in-depth RNA sequencing to assemble a comprehensive dataset of gene expression profiles from 24 unique types of human and mouse lung, lymph node macrophages, human blood and mouse spleen macrophages. Only 130-230 genes of the top 1,000 marker genes, are shared between human and mouse macrophage populations (Leach et al., 2020). Understanding the differences between mouse and human macrophages is detrimental as different results may arise due to the different species.

**Figure 2:**
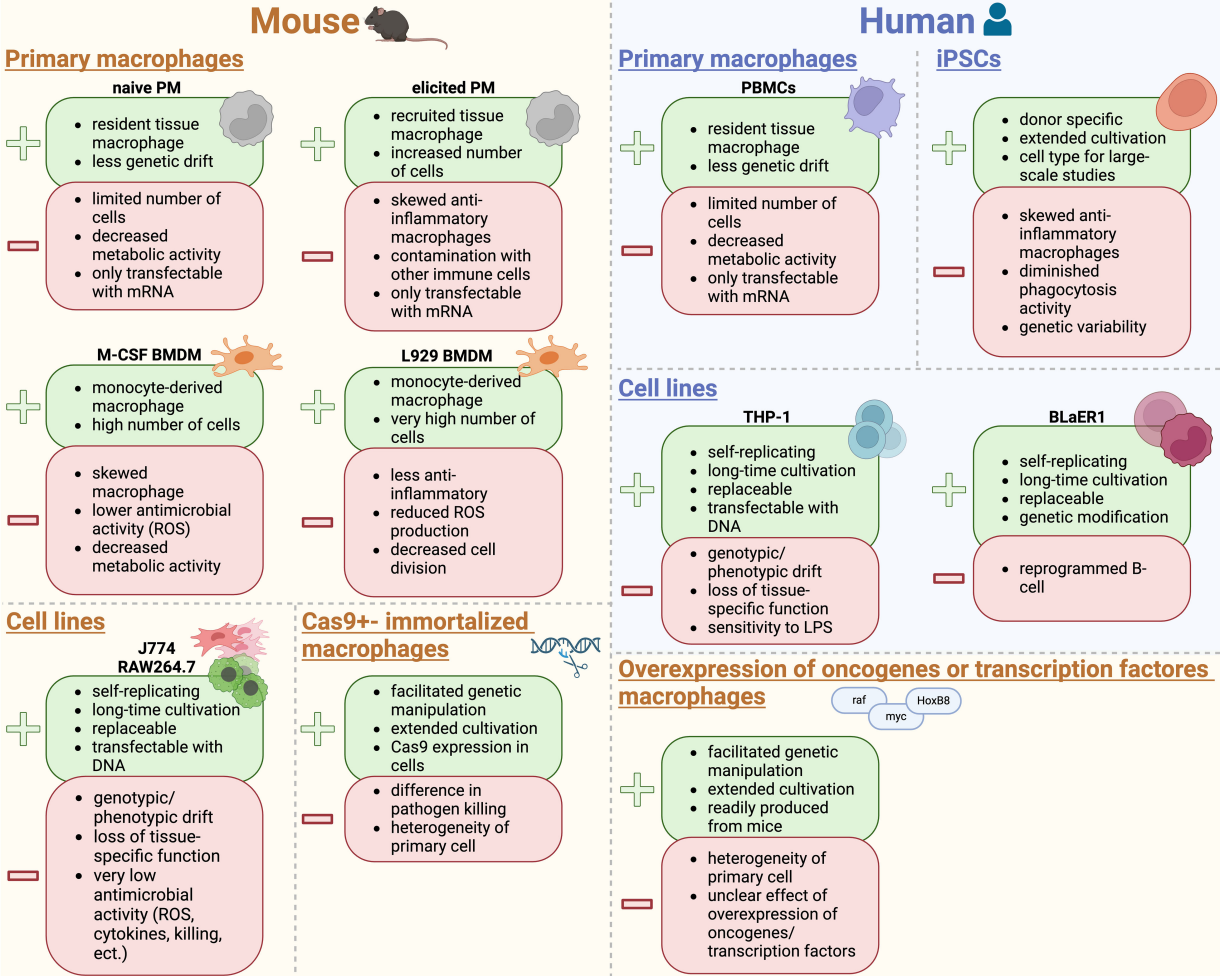
Advantages and disadvantages of mouse and human primary cells, commonly used cell lines, as well as, genetically modified cells. Figure created in BioRender.com. PM, peritoneal macrophages; BMDM, bone marrow-derived macrophages; M-CSF, macrophage colony-stimulating factor; ROS, reactive oxygen species; PBMCs, peripheral blood monocytes; iPSCs, induced pluripotent stem cells; LPS, lipopolysaccharide.

**Figure 2** provides a summary of the advantages and disadvantages of the most commonly used mouse and human macrophage types.

### 8.4 Macrophages from other species

One species that gives rise to various macrophage cell lines is chicken. Similar to thioglycollate elicited PMs in mice, inflammatory macrophages in the peritoneum of the chicken are recruited with Sephadex as a stimulant (Trembicki et al., 1984; Qureshi et al., 2000). These macrophages can efficiently phagocytose and break down bacteria through lysosomal acid hydrolysis (Qureshi et al., 2000). Immortalized chicken macrophage cell lines offer an extra resource for macrophage studies.

Chicken macrophage cell lines, like MQ-NCSU (Qureshi et al., 1990) and HD11 (Beug et al., 1979), are widely used in studies. HD11, derived from chicken bone marrow and transformed with avian myelocytomatosis virus type MC29, exhibits macrophage-like characteristics, including enhanced morphology and ROS production (Graf, 1973; Wisner et al., 2011). MQ-NCSU, from a JM/102W strain of Marek’s disease virus-infected broiler-type chicken spleen, shows traits of malignancy and mononuclear phagocyte lineage (Qureshi et al., 1990). Even primary monocytes can be isolated from heparinized blood obtained from the wing vein of chickens (Wigley et al., 2002, 2006).

Moreover, macrophages originating from bovine tissue are commonly used for *in vitro* models. Monocyte-derived macrophages from the peripheral blood of cows are used for *in vitro* infection experiments (Liebana et al., 2000; Weiss et al., 2002; Janagama et al., 2006), macrophage polarization studies (Imrie and Williams, 2019) and cytokine production (Werling et al., 2004). Moreover, alveolar macrophages harvested by pulmonary lavage were analyzed for immune response against pathogens (Widdison et al., 2007, 2011). In addition, BMDM generated from the iliac crest of bovines are infected with *Mycobacterium bovis* and *Mycobacterium avium subsp. paratuberculosis* strains to identify potential diagnostic biomarkers by analyzing the cytokine production and gene expression levels (Amato et al., 2024).

## 9 Conclusion

Macrophages represent one of the most plastic and versatile cell types present in multicellular organisms, characterized by their vast possibilities of signal recognition, cellular adaptations in terms of metabolism, their receptor repertoire, and the array of produced and secreted substances, as well as various forms of phagocytosis and endocytosis. Due to their presence in nearly all body tissues or fluids, macrophages constantly detect and respond to environmental shifts as well as tissue physiology, adapting their functional and metabolic states accordingly. The choice of cell type depends on the specific questions being posed. Consequently, in studies investigating macrophage functions and behaviors, it is crucial to select a macrophage type that closely resembles the *in vivo* setting in order to accurately represent the tissue macrophage type being investigated.

However, this approach is often highly challenging in terms of isolation and cultivation of primary macrophage types. Variables such as cell culture media, serum, and cell culture dishes can affect macrophage function, so a necessary understanding of their potential effects needs to be considered.

These observations underscore the need for caution when interpreting *in vitro* studies, as they may not always accurately reflect *in vivo* phenotypes. Therefore, it is essential to validate findings from macrophage-like cell lines with studies involving primary macrophages to accurately characterize macrophage functions. This is especially relevant for results significant to humans, indicating the use of human macrophage cell lines, as human macrophages exhibit specific reactions and interactions that may differ from those of mouse macrophage cell lines.
