# Corrigendum: Macrophage variants in laboratory research: most are well done, but some are RAW

**DOI:** 10.3389/fcimb.2025.1575550

**Published:** 2025-02-26

**Authors:** Marc Herb, Valentin Schatz, Karina Hadrian, Deniz Hos, Bohdan Holoborodko, Jonathan Jantsch, Natascha Brigo

**Affiliations:** ^1^ Institute for Medical Microbiology, Immunology and Hygiene, Faculty of Medicine and University Hospital Cologne, University of Cologne, Cologne, Germany; ^2^ Department of Ophthalmology, Faculty of Medicine and University Hospital Cologne, University of Cologne, Cologne, Germany; ^3^ Institute of Clinical Microbiology and Hygiene, University Hospital Regensburg and University of Regensburg, Regensburg, Germany

**Keywords:** macrophage types, primary macrophages, macrophage-like cell lines, immunity, macrophage polarization (MP)

In the published article, there was an error in the **Funding** statement. The funding information for M.H. was incorrect. Original text: “The author(s) declare financial support was received for the research, authorship, and/or publication of this article. JJ received funding from the DFG TRR 374 grant (project No. 509149993, TP B05), DFG CRC 1607 grant (project No. 501530074, TP B02) and DFG Research Training Group 2740 Immunomicrotope. DH received funding from the DFG CRC1607 (TP B02).” The position of MH was funded by grants from the state of North-Rhine-Westphalia, Germany, (AZ: 323-8.04.10.02-141905), German Center for Infection Research, DZIF (TTU 08.927 and TTU 08.928) and the Deutsche Forschungsgemeinschaft (DFG), SFB 670 procured by Prof. Dr. Martin Krönke. The correct Funding statement appears below.

“The author(s) declare financial support was received for the research, authorship, and/or publication of this article. JJ received funding from the DFG TRR 374 grant (project No. 509149993, TP B05), DFG CRC 1607 grant (project No. 501530074, TP B02) and DFG Research Training Group 2740 Immunomicrotope. DH received funding from the DFG CRC1607 (TP B02).”

The authors apologize for this error and state that this does not change the scientific conclusions of the article in any way. The original article has been updated.

